# Structural characteristics of research ethics committees in Japan and their relevance to regulatory science: a nationwide descriptive study

**DOI:** 10.1186/s12910-026-01553-w

**Published:** 2026-07-07

**Authors:** Tomohiko Hara, Yuki Iwahara, Shunsaku Murakawa

**Affiliations:** Office of Research Administration, Center for Regulatory Science, Pharmaceuticals and Medical Devices Agency, Tokyo, Japan

**Keywords:** Ethics committee, Ethics, Professional competence, Government regulation, Regulatory science, Epidemiology, Registry, Japan

## Abstract

**Background:**

Research ethics committees (RECs) and institutional review boards provide independent ethical oversight of health-related research. In Japan, the Pharmaceuticals and Medical Devices Agency (PMDA) increasingly conducts regulatory science (RS) research, which aims to generate scientific evidence to support regulatory decision-making. However, nationwide REC structures in Japan, and their alignment with the needs of RS oversight, have not been described.

**Methods:**

We conducted a nationwide cross-sectional descriptive study using publicly available data from the Research Ethics Review Committee Reporting System of Japan (June 5, 2024). Committee size, gender composition, and members’ professional expertise were analyzed by institution type. We compared these findings with structural requirements in Japanese regulations and guidelines. In addition, we conducted a complementary literature-based analysis of PMDA-affiliated RS research using PubMed. Major Medical Subject Headings (MeSH) terms were examined to identify dominant research areas and to contextualize the REC structure within the current landscape of RS research.

**Results:**

A total of 2,604 RECs comprising 26,786 members were identified nationwide. The mean committee size was 10.3. Overall, committees commonly included multiple lay members, with 68.0% of institutions having at least two public representatives. In contrast, a substantial gender imbalance was observed, with 70.1% of committee members being male across all institutional categories. Committee composition varied by institution type. Analysis of MeSH terms in PMDA-affiliated RS research publications showed frequent focus on regulatory review, pharmacovigilance, advanced biomedical technologies, and clinical trial methodology.

**Conclusions:**

Japanese RECs were characterized by variation in committee size, occupational composition, and lay representation, while substantial gender imbalance persisted. Variations in committee composition reflected institutional differences in research characteristics. In the context of RS research, REC structures that incorporate relevant expertise alongside diverse perspectives may help inform discussions on ethical review practices.

**Supplementary Information:**

The online version contains supplementary material available at https://doi.org/10.1186/s12910-026-01553-w.

## Background

Maintaining transparency in research ethics is essential for ensuring research integrity. Research ethics committees (RECs) and institutional review boards (IRBs) [1] provide independent ethical review of health-related research, including consideration of scientific validity relevant to ethical acceptability, as emphasized in major international guidelines, including the World Medical Association’s Declaration of Helsinki [2], the International Council for Harmonisation [3], the World Health Organization [4], and the Council for International Organizations of Medical Sciences [5].

Despite national variations, international guidance generally requires RECs/IRBs to include members with scientific, ethical, and legal expertise, as well as the general public representatives (lay members), and to consult independent experts when appropriate. International guidance also emphasizes committee diversity, taking into account factors such as age, gender, and race or ethnicity [6]. Requirements vary across countries regarding expertise, lay representation, and gender balance [7]. In the United States (U.S.), these requirements are stipulated by the Common Rule [8, 9] and U.S. Food and Drug Administration (FDA) regulations [10].

In Japan, committees reviewing clinical trials under the Good Clinical Practice Ordinance are often referred to as IRBs [3]. Certified review boards (CRBs) review specified clinical research under the Clinical Research Act [11], while committees reviewing research under national ethical guideline are generally referred to as RECs [12]. Ethical review requirements are defined across multiple laws and guidelines rather than a single framework. Among these, the Ethical Guidelines for Medical and Biological Research Involving Human Subjects serve as a foundational framework for REC establishment nationwide, including representatives of the general public and independent specialists, such as medical and healthcare professionals, as well as experts in ethics and law [13]. Under these guidelines, at least two committee members must be unaffiliated with the organizing institution. Furthermore, the REC must have at least five members, including both male and female members [12, 13]. The composition and characteristics of RECs in Japan are publicly reported through the Research Ethics Review Committee Reporting System of the National Institute of Public Health, currently available on the website of the Ministry of Health, Labour and Welfare (MHLW) [14]. Previous analyses of RECs/IRBs registration databases, including those in the United States, have shown that such databases can be used to characterize the overall IRB landscape and basic demographic features, such as committee composition and member characteristics [15].

Regulatory authorities, such as the U.S. FDA [16], the European Medicines Agency [17], and the Pharmaceuticals and Medical Devices Agency (PMDA) [18], actively conduct Regulatory Science (RS) research. RS is a multidisciplinary field focused on developing scientific tools, standards, and methodologies for assessing the quality, safety, and efficacy of medicinal and healthcare products to inform regulatory decision-making. As part of its 5th Mid-term Plan (2024–2029), PMDA aims to promote RS research, and the number of related research publications has increased in recent years. Such research contributes to regulatory decision-making by integrating knowledge from basic, clinical, and social sciences [19–22]. Recent advances, such as new biotechnology, big data [23], real-world data science [24, 25], and generative artificial intelligence (AI) [26, 27], have diversified the types of RS research approaches. RS research conducted by PMDA is subject to external review to ensure adherence to key principles, such as scientific rigor, ethical integrity, validity, fairness, accountability, confidentiality, and transparency [28]. These advances have drawn attention to the need for understanding how RECs are structured to review RS research within evolving scientific, ethical, and regulatory contexts.

While general ethical principles and national guidelines provide a basic framework for the ethical review of research, there are no clearly defined standards specifying REC composition for reviewing RS research conducted by PMDA. Given the diverse and interdisciplinary nature of RS research, examining how RECs are currently structured across Japanese institutions, including government organizations, and considering the types of current RS research may provide useful context for understanding existing practices. In this study, we describe nationwide REC structures in Japan by comparing registry data with regulatory requirements and contextualize them using PMDA-affiliated RS publications.

## Methods

### Ethical considerations

This study analyzed only publicly available information and did not constitute health-related research involving human participants. Therefore, informed consent and ethics review were not required under the Japanese Ethical Guidelines for Medical and Biological Research Involving Human Subjects [13]. Artificial intelligence tools were used solely for language editing and to improve the clarity of the English manuscript under the full supervision of the authors.

### Review of Japanese regulations and guidelines

In Japan, the MHLW and the Ministry of Education, Culture, Sports, Science and Technology (MEXT), and the Ministry of Economy, Trade and Industry are involved in the regulation of biomedical research. Regulatory requirements were assessed at the time of data extraction on June 5, 2024 and subsequently re-checked on March 31, 2026 through searches of the MHLW [11, 29, 30] and MEXT [31] websites, confirming that no major updates affecting the structural requirements of RECs/IRBs/CRBs had occurred. We used this information about ethical guidelines and acts to identify the requirements for RECs/IRBs/CRBs in Japan. We used the retrieved documents to extract information about the minimum number of committee members, gender specifications, stipulated areas of expertise, and the necessity for independent expert consultants.

### Data collection and processing

In this nationwide cross-sectional descriptive study, we collected data from the Japanese database of the Research Ethics Review Committee Report System [14] (Data accessed June 5, 2024). Units of analysis were RECs and member attributes recorded in the registry. We extracted information about the institutions and ethics committee members, including the gender and occupation, which are self-reported by institutions. The attributes of each REC member were recorded according to the Japanese Ethical Guidelines for Medical and Biological Research Involving Human Subjects as follows: 1) experts in natural sciences, 2) public representatives, 3) experts in humanities and social sciences research, and 4) others. These role categories are standardized within the registry system.

After data cleaning, the extracted data were categorized by institution type and member occupation. Subsequently, we reclassified and aggregated the occupations into specific fields of expertise based on related keywords identified during data processing using a predefined keyword-based classification (Supplemental Table 1). The occupations were broadly classified into three categories: 1) science experts, 2) humanities and social sciences researchers, and 3) the general public. Science experts were classified as biomedical/human research or nonhuman research. Biomedical/human and humanities/social research were further grouped into major specialties (Supplemental Table 1). Experts in biomedical and human research included a broad range of health-related disciplines, including physicians and dentists, nursing and allied health professionals, pharmaceutical sciences, genetics and molecular biology, medical devices and engineering, epidemiology/statistics, and clinical research. The nonhuman research included fields such as engineering, information and computational sciences, chemical and material sciences, agricultural and food sciences, and environmental and ecological sciences. Institutions were classified into 10 categories: hospital, university, company, clinic, nonprofit organization, research institute, medical corporation, academic society, government, and national center. When committee members held multiple professional roles, each role was counted separately. Occupational distributions reflect expertise counts, not unique individuals. Members categorized as the general public were defined as not belonging to any of the specified occupational categories. When a committee member was affiliated with more than one organization, this was designated as a distinct case.

### Statistical analysis

We used open-source R version 4.3.3 (R Foundation for Statistical Computing) [32] and Microsoft Excel (Redmond, WA, USA) for data processing, analysis, and visualization. We generated heatmaps using the R packages “ggplot2” and “ComplexHeatmap” [33]. We performed binomial tests to compare the male-to-female ratios, assuming a ratio of 1:1. A *p*-value < 0.05 was considered statistically significant for all tests. The proportion data were analyzed using hierarchical and nonhierarchical clustering methods, which group institutions with similar REC compositions, thereby enabling the identification of structural patterns across different types of institutions. The default method of hierarchical clustering was performed using Euclidean distance. We determined the category in the hierarchical cluster classification, and the hierarchical classification was set to six based on the dendrogram distances. In the dendrogram, we identified the cut-off height at which the increase in within-cluster variance was most pronounced. For nonhierarchical clustering, we used the k-means algorithm with Euclidean distance.

### Literature search strategy for RS research in PMDA

This literature-based analysis was conducted to characterize the thematic scope of RS research affiliated with the PMDA as a complementary analysis. A comprehensive literature search (data accessed December 14, 2025) was conducted using PubMed to identify all publications affiliated with the PMDA of Japan. The search was performed by specifying PMDA-related institutional names in the affiliation field (e.g., “Pharmaceuticals and Medical Devices Agency”). No restrictions were applied regarding publication year, study design, or research field. For each identified publication, bibliographic information and Medical Subject Headings (MeSH) terms assigned by the National Library of Medicine were extracted. Among the extracted MeSH terms, MeSH major topic terms were used for subsequent thematic analysis to capture the core research focus of the publications. The most frequently occurring MeSH major topic terms were interpreted as representing the dominant research areas addressed in PMDA-affiliated RS research publications.

## Results

### Japanese regulations and guidelines for ethical review bodies

We evaluated 14 Japanese research ethics–related regulations and guidelines listed on official government websites [3, 11, 29–31]. In Japan, the composition of ethical review bodies are specified by these regulatory frameworks. Most guidelines require a minimum of five members, while certain specialized committees, such as certified committees for high-risk regenerative medicine, require eight or more members (Table 1).

**Table 1:** Requirements for ethical review bodies according to Japanese guidance documents

Guidance document	Minimum committee composition number (by gender*)	Required expertise	Expert consultation
Ethical Guidelines for Medical Research Involving Human Subjects^a)^	5 (1)	Natural sciences; humanities/social sciences; general public	Expert opinion or advice possibly required
Act on Securing Quality, Efficacy and Safety of Products Including Pharmaceuticals and Medical Devices^b)^	5 (1)	Medical professionals; general public	Expert committee or expert opinion/advice possibly required
Act on the Safety of Regenerative Medicine^c)^	8 (2)	Expert in molecular/cell biology, genetics, clinical pharmacology, or pathology; regenerative medicine; clinical medicine; cell culture processing; law; bioethics; biostatistics; general public	Technical expert required
Clinical Trials Act^d)^	5 (1)	Medicine/healthcare; law/bioethics; general public	Technical expert required
Guidelines on the Derivation of Human Embryonic Stem Cells^e)^	5 (2)	Natural sciences; humanities/social sciences; general public	Not stated
Guidelines for the Distributing Institute of Human Embryonic Stem Cells^e)^	5 (1)	Natural sciences; humanities/social sciences; general public	Not stated
Guidelines on the Utilization of Human Embryonic Stem Cells^e)^	5 (1)	Natural sciences; humanities/social sciences; general public	Not stated
Guidelines on the Research on Producing Germ Cells from Human iPS Cells or Human Tissue Stem Cells^e)^	5 (2)	Medicine; biology; law; bioethics; general public	Not stated
Ordinance for Enforcement of the Act on Regulation of Human Cloning Techniques/Guidelines on the Handling of Specified Embryos^f)^	5 (2)	Natural sciences; humanities/social sciences; general public	Not stated
Ethical Guidelines for Research That Involves the Creation of Human Fertilized Embryos^g)^	6 (2)	Reproductive medicine; biology; law; bioethics; general public; genetic engineering technology	Expert opinion or advice possibly required
Ethical Guidelines for Research Using Gene-altering Technologies on Human Fertilized Embryos^h)^	5 (2)	Reproductive medicine; genetic engineering technology; humanities/social sciences; bioethics; general public	Expert opinion or advice possibly required
Ethical Guidelines for Clinical Trials on Gene Therapy^i)^	5 (1)	Expert in molecular/cell biology, genetics, clinical pharmacology or pathology; clinical medicine; law; bioethics; biostatistics; general public	Expert opinion or advice possibly required
Guidelines for Infectious Disease Issues in Public Health Associated with the Implementation of Heterologous Transplantation^j)^	5 (1)	Natural sciences; humanities/social sciences; general public; basic medical science of infectious diseases; clinical infectious diseases; infectious disease laboratory science; infectious disease epidemiology; public health administration; veterinary medicine	Expert opinion or advice possibly required
Guidelines for animal experiments conducted under the Jurisdiction of the Ministry of Health, Labour and Welfare^k)^	3 (0)	Expert in animal experiments; laboratory animals; science	Not stated

The minimum number of persons required includes at least one of each gender

https://www.mhlw.go.jp/content/001077424.pdf

https://www.pmda.go.jp/files/000230974.pdf

https://www.mhlw.go.jp/stf/seisakunitsuite/bunya/0000150542_00001.html

https://www.mhlw.go.jp/stf/seisakunitsuite/bunya/0000163417.html

https://www.mext.go.jp/a_menu/lifescience/bioethics/hito_es.html

https://www.mext.go.jp/a_menu/lifescience/bioethics/clone.html

https://www.mext.go.jp/seisyoku_hojo.html

https://www.mext.go.jp/a_menu/lifescience/bioethics/embryoediting.html

https://www.mhlw.go.jp/content/001077219.pdf

https://www.mhlw.go.jp/content/001410327.pdf

https://www.mhlw.go.jp/file/06-Seisakujouhou-10600000-Daijinkanboukouseikagakuka/honbun.pdf

These regulations generally mandate inclusion of members with expertise in medicine or healthcare, ethics or law, and the natural or social sciences, as well as at least one lay member. Many guidelines also require at least one male and one female member, ensuring minimum gender representation. Some guidelines further differentiate the necessary expertise depending on the type of research reviewed, such as regenerative medicine, gene therapy, or embryo research. Regarding the involvement of external experts, all major Japanese regulations, including the Ethical Guidelines for Medical and Biological Research, the Clinical Trials Act, and the Act on the Safety of Regenerative Medicine, allow ethical review bodies to seek advice from external specialists when needed. These frameworks share a common principle: committees may obtain expert input to ensure appropriate review of highly specialized or technically complex research.

### Structural features of RECs in Japan

We identified 2,604 RECs with a total of 26,786 members and classified them by institution type (Table 2). Most RECs were in hospitals (1,146 committees, 44.0%), followed by universities (481 committees, 18.5%), whereas only 32 committees (1.2%) were at government institutions.

**Table 2:** Number of RECs (N) and members (n) in Japan categorized by institution type

Institution	N (%)	n	n/N	Men* n^m^ (%)	Women* n^w^ (%)
Total	2,604	26,786	10.3	18,767 (70.1)	8,019 (29.9)
Hospital	1,146 (44.0)	12,826	11.2	9,422 (73.5)	3,404 (26.5)
University	481 (18.5)	5,747	11.9	3,898 (67.8)	1,849 (32.2)
Company	271 (10.4)	2,087	7.7	1,397 (66.9)	690 (33.1)
Clinic	231 (8.9)	1,765	7.6	1,036 (58.7)	729 (41.3)
Nonprofit Organization	186 (7.1)	1,653	8.9	1,137 (68.8)	516 (31.2)
Research Institute	94 (3.6)	886	9.1	612 (69.1)	274 (30.9)
Medical Corporation	94 (3.6)	796	8.5	528 (66.3)	268 (33.7)
Academic Society	60 (2.3)	536	8.9	395 (73.7)	141 (26.3)
Government	32 (1.2)	326	10.2	222 (68.1)	104 (31.9)
National Center	9 (0.4)	164	18.2	120 (73.2)	44 (26.8)

^*^Binomial tests revealed statistically significant gender disparities (*p* < 0.001) among all institutional categories

N: number of institutes, n: number of members, n^m^: number of male members, n^w^: number of female members

Mean committee size was 10.3 ± 4.2. National centers had the largest committees, with a mean of 18.2 ± 7.8 members. Institution-level comparisons showed that the mean number of committee members in government institutions (10.2 ± 3.1) was lower than in hospitals (11.2 ± 3.3) and universities (11.9 ± 5.8) but higher than in companies (7.7 ± 3.2) and medical corporations (8.5 ± 3.2).

Table 2 summarizes the gender composition of ethics committee members (*n* = 26,786) across different institution categories in Japan. Overall, 70.1% (18,767 members) were male and 29.9% (8,019 members) were female (*p* < 0.001). The highest proportions of male members were observed in hospitals (73.5%), academic societies (73.7%), and national centers (73.2%). In contrast, clinics had the highest proportion of female members (41.3%), followed by the government institutions (31.9%). Although all committees fulfilled the national regulatory requirement of including at least one male and one female member, the overall distribution showed substantial gender imbalance across all institution categories. Binomial tests confirmed that this gender disparity was statistically significant in every category (*p* < 0.001).

Regarding the professions of ethics committee members as originally reported in the national registry, 56.9% (15,252 members) were science experts, followed by members of the general public (19.0%, 5,089 members), experts in humanities and social sciences research (17.5%, 4,698 members), and others (6.5%, 1,747 members). For subsequent analyses, reported occupations were reclassified and aggregated into defined categories. Table 3 presents a classification of the members’ professions according to their areas of expertise and the gender distribution. The largest occupation category was physicians and dentists (26.2%, 7,013 members), the majority of whom were males (males: females = 6,191: 822). The second largest category was the general public (22.3%, 5,984 members), with a slightly more balanced gender distribution (males: females = 4,046: 1,938). Regarding nurses and midwives (10.7%, 2,859 members), 86.7% were female (males: females, 379: 2,480). Many committees included more than one lay member; specifically, 1,771 of 2,604 institutions (68.0%) had at least two.

**Table 3:** Distribution of occupation categories among RECs members

OCCUPATION	TOTAL (%) (*N* = 26,786)	MEN (*N* = 18,767)	WOMEN (*N* = 8,019)
Physicians and dentists	7,013 (26.2%)	6,191	822
General public	5,984 (22.3%)	4,046	1,938
Nursing and midwifery	2,859 (10.7%)	379	2,480
Legal and judicial affairs	2,210 (8.3%)	1,768	442
Pharmaceutical sciences and industry	2,194 (8.2%)	1,651	543
Psychology, religion, and ethics	1,245 (4.6%)	844	401
Co-medical and allied health professionals	1,202 (4.5%)	744	458
Humanities and social research (other)	927 (3.5%)	606	311
Nongenomic human biology and related fields	905 (3.4%)	753	152
Experts in nonhuman science	797 (3.0%)	629	168
Diagnostics (in vivo and in vitro)	787 (2.9%)	580	207
Genetics, genomics, and molecular biology	613 (2.3%)	496	117
Social medicine and public health	546 (2.0%)	419	127
Medical devices and engineering	340 (1.3%)	296	44
Epidemiology and statistics	310 (1.2%)	213	97
Clinical trials and research	80 (0.3%)	58	22
Reproductive medicine and biology	69 (0.3%)	48	21
Toxicology and veterinary sciences	40 (0.1%)	36	4

Given the possibility that an individual may have multiple occupations, a degree of overlap exists among the values

**Fig. 1:**
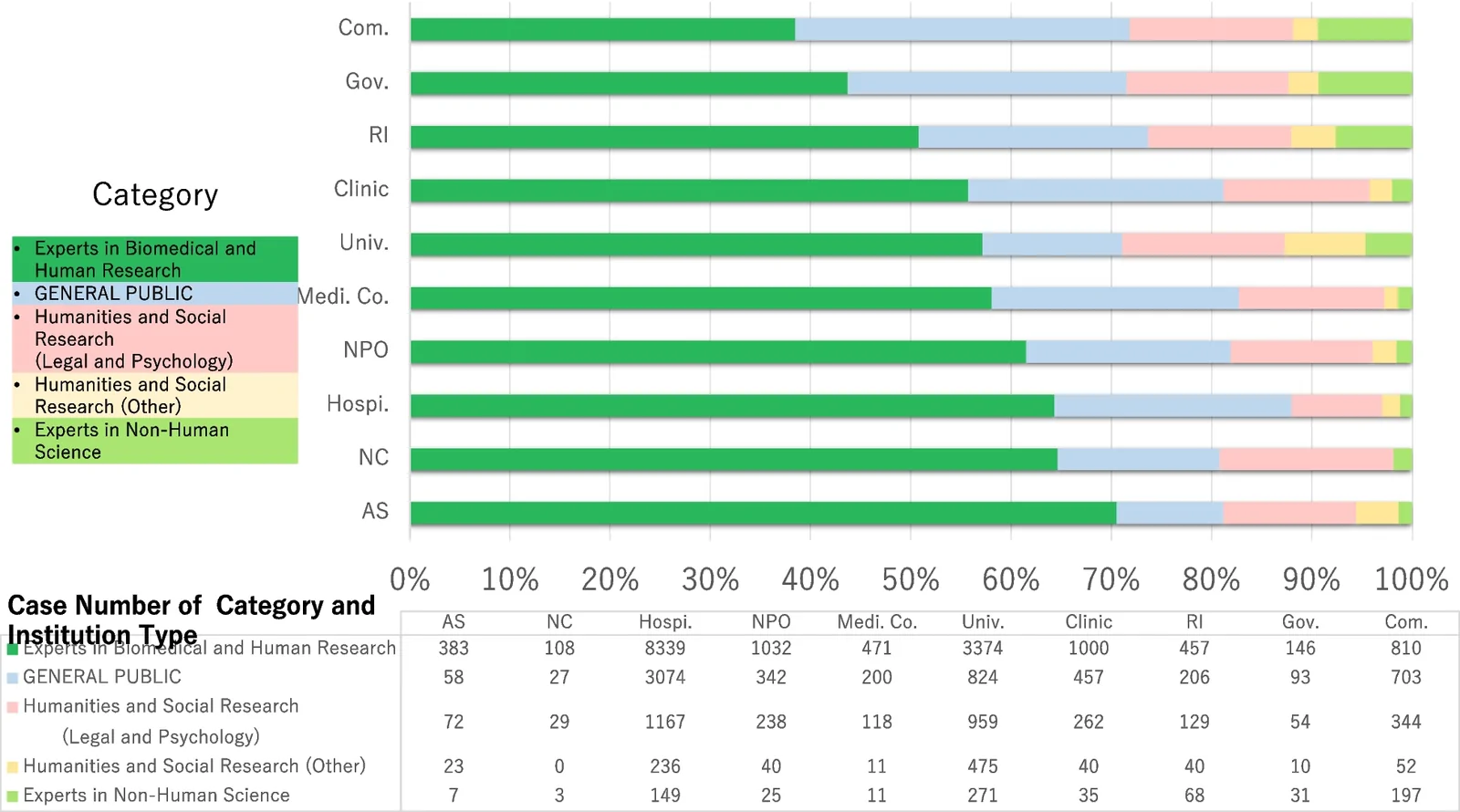
Proportional Comparison of Occupation Categories Across Institution Categories. Com., company; Gov., government; RI, research institute; Univ., university; Medi. Co., medical corporation; NPO, nonprofit organization; Hospi., hospital; NC, National Center; AS, academic society

Figure 1 shows the key differences of the occupation categories among the 10 institutional categories according to their frequency distribution, highlighting that institutional types differ substantially in the balance between scientific, public, and humanities-related expertise. Supplemental Table 2 provides a more detailed breakdown. Experts in biomedical and human research were most prominent in RECs at academic societies (71.5%), national centers (65.9%), and hospitals (65.0%), whereas government institutions showed a lower proportion (44.8%). General public participation was highest in companies (33.7%), followed by government institutions (28.5%) and clinics (25.9%). Meanwhile, humanities experts, particularly those in legal and ethical roles, were prominent in national centers (17.7%), universities (16.7%), and government institutions (16.6%). Physicians and dentists were concentrated in academic societies (40.3%), hospitals (33.7%), and clinics (27.8%), with government institutions accounting for 12.9%. Nursing and midwifery professionals were highest in hospitals (12.8%), and universities (12.5%) but were limited in government institutions (8.0%). Experts in pharmaceutical sciences and the pharmaceutical industry were prominent in nonprofit organizations (18.1%), and companies (13.0%) but minimally represented in government institutions (4.6%). Government institutions exhibited a distribution of member roles that was less concentrated in specific professional categories compared with medical institutions, reflecting a comparatively broader range of expertise and backgrounds.

**Fig. 2:**
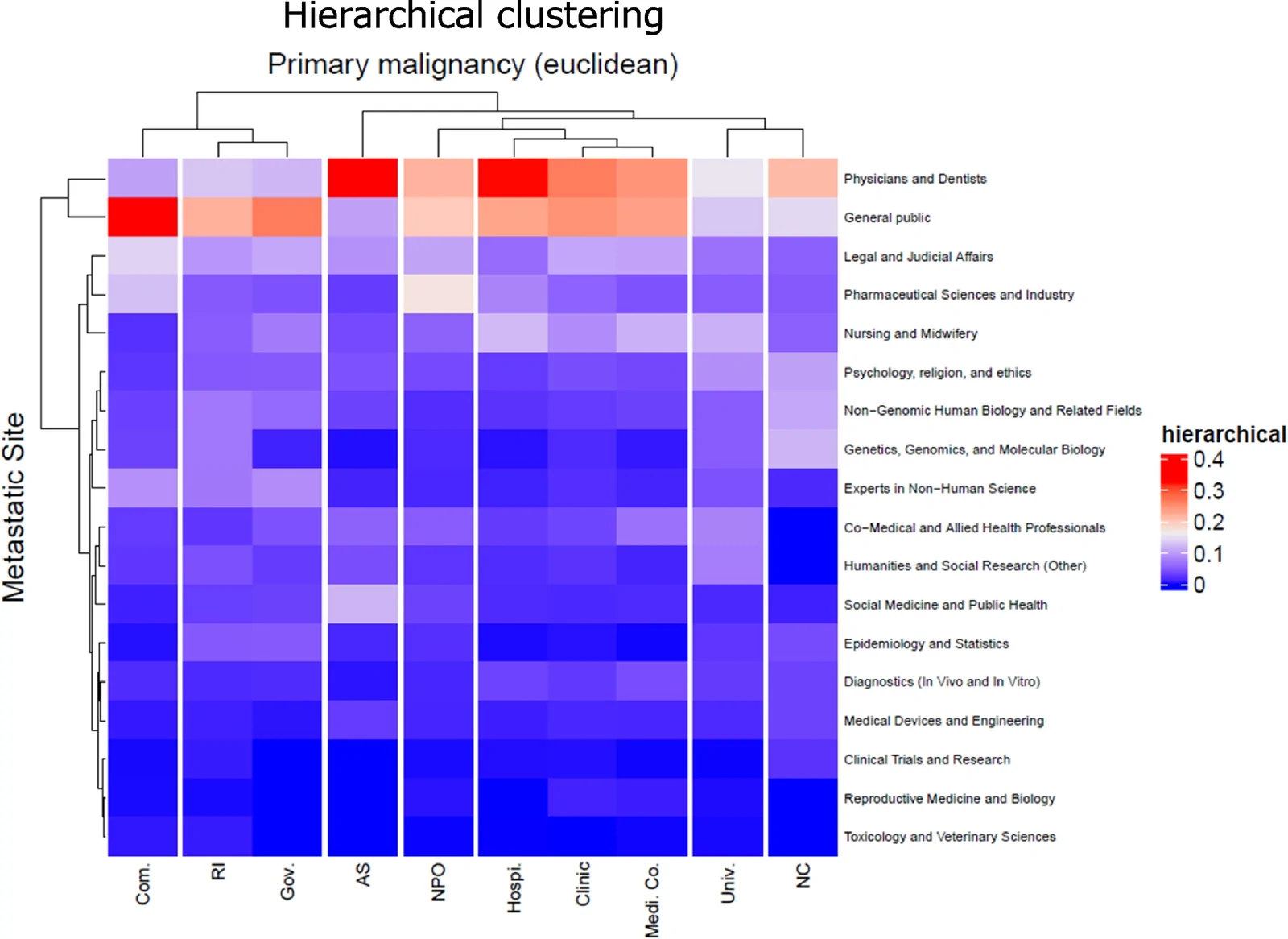
Hierarchical Clustering of Occupational Composition Ratios Across Institution Categories. Determination of the category in the hierarchical cluster classification was confirmed using forward selection. The hierarchical classification was set to six based on the dendrogram distances. Com., company; RI, research institute; Gov., government; AS, academic society; NPO, nonprofit organization; Hospi., hospital; Medi. Co., medical corporation; Univ., university; NC, National Center

Figure 2 presents a hierarchical cluster classification illustrating similarities in occupational composition ratios across institutions, demonstrating that medical institutions cluster together, whereas academic societies and government-related institutions show distinct compositional patterns. Government-related institutions showed trends comparable to those of research facilities and companies. This similarity was also observed in the nonhierarchical cluster classification (Supplemental Fig. 1).

### RS research in the PMDA

A total of 456 articles affiliated with the PMDA were identified through a PubMed search. From these publications, 577 unique MeSH major topic terms were extracted, yielding 1,116 assignments in total. The most frequently assigned MeSH major topic was *Drug Approval* (*n* = 52), followed by *Clinical Trials as Topic* (*n* = 23) and *Antineoplastic Agents* (*n* = 22).

Among MeSH major topics appearing more than five times (Supplemental Table 3), many were related to research areas requiring specialized expertise. Frequently observed topics included those related to regulatory review, such as *Drug Approval*, *Device Approval*, *Guidelines as Topic*, and *Drug Industry*. Topics associated with pharmacovigilance were also common, including *Drug-Related Side Effects and Adverse Reactions*, *Adverse Drug Reaction Reporting Systems*, *Drug Labeling*, *Drug and Narcotic Control*, and *Product Surveillance, Postmarketing*. In addition, several topics reflected advanced biomedical technologies, such as *Biological Products*, *Cell- and Tissue-Based Therapy*, *Regenerative Medicine*, and *Genetic Therapy*, along with MeSH major topics related to clinical trial methodology, including *Clinical Trials as Topic*, *Randomized Controlled Trials as Topic*, *Clinical Trials, Phase I as Topic*, *Multicenter Studies as Topic*, and *Research Design*, which were frequently assigned.

## Discussion

In Japan, RS research conducted by the PMDA is subject, where applicable, to the Ethical Guidelines for Medical and Biological Research Involving Human Subjects, which require review by RECs. In addition, advisory committees with members from academia, industry, and the public oversee aspects such as conflict of interest management, research integrity, and public disclosure through established frameworks. In this context, understanding the structural characteristics of RECs at a national level provides an important benchmark for considering committee composition in RS-related ethical governance.

We compared nationwide registry data on Japanese REC structures with regulatory requirements. Overall, RECs had a mean size of 10.3 members and commonly included multiple lay members. Despite meeting gender inclusion requirements, committee composition was markedly imbalanced, with 70.1% of members being male across all institution categories. These findings provide a national baseline description of REC composition in Japan and may serve as a reference point for future evaluations of governance, diversity, and expertise. The observed disparities may be consistent with broader structural gender patterns reported in Japanese scientific and healthcare institutions [34]. Moreover, formal compliance with diversity requirements does not necessarily translate into substantive representation. From an ethical perspective, diversity is understood not only as a matter of fairness but also as a means of incorporating a plurality of perspectives that may enhance deliberative quality and legitimacy [35]. In this context, imbalances in gender representation and professional composition—such as the predominance of science experts (56.9%)—as well as disparities in participation and influence, may constrain the integration of diverse viewpoints into decision-making.

Committee composition differed across institution types. This variability likely reflects differences in institutional size, mission, and the typical research portfolios of each institution. Given that RS research of the PMDA often involves social, administrative, and regulatory considerations in addition to scientific assessment, the diversity observed in government institution committees may provide useful contextual insight when reflecting on REC structures relevant to RS research review.

We additionally conducted a complementary literature-based overview of RS research affiliated with the PMDA using MeSH major topics. The MeSH analysis was intended to provide a thematic overview of RS-related research rather than a formal evaluative assessment. The thematic profile of PMDA-affiliated publications suggested frequent coverage of areas such as regulatory review, pharmacovigilance, advanced biomedical technologies, and clinical trial methodology, highlighting the relevance of expertise in regulatory, clinical, biomedical, and biostatistical fields.

The major frameworks generally allow committees to seek advice from external specialists. The expertise of standing committee members depends on the area of specialization of the content reviewed. An REC in regenerative medicine requires technical experts in eight different fields of specialization and related diseases (molecular/cell biology, genetics, clinical pharmacology or pathology; regenerative medicine; clinical medicine; cell culture processing; law; bioethics; biostatistics, and the general public), and the fact that experts in clinical research methodology are required is a distinctive feature. Institutional Animal Care and Use Committees are recommended to consist of laboratory animal science experts, public representatives, clinical veterinarians, clinical study experts [36], experts in regulatory compliance, nonscientist representatives, and other specialists if required [37].

This study has several limitations. First, because the analysis was limited to describing the structural features of Japanese RECs, it does not allow conclusions about the ideal or most appropriate composition for RECs. Second, the nationwide dataset reflects available administrative records, which limit the depth of information on committee functions, operational practices, and actual review activities, including meeting-level participation. Therefore, operational aspects of REC review processes could not be evaluated, and the findings should be interpreted cautiously. Registry-based REC data are not primarily designed for analytical or benchmarking purposes and may lack standardized and validated information, limiting analyses largely to basic demographic characteristics [15]. Third, the dataset covers only a single year, preventing any examination of temporal trends or changes in REC composition over time. Finally, although the RS literature analysis describes the thematic distribution of PMDA-affiliated publications and highlights the relevance of REC member expertise for RS research, it does not draw conclusions regarding an ideal RS-specific committee composition.

## Conclusion

In this nationwide descriptive study, RECs in Japan were characterized by variation in committee size, occupational composition, and lay representation, while substantial gender imbalance persisted across institution types. Committee composition varied by institutional category, reflecting differences in organizational missions and research portfolios. Together, these findings and the thematic distribution of RS research provide descriptive insight into structural characteristics of RECs relevant to the ethical review of RS research. Continued attention to diversity and breadth of expertise may inform future discussions on the organization and potential refinement of REC structures in response to increasingly complex and interdisciplinary research environments.

## Supplementary Information

Supplementary Material 1.

## Data Availability

The datasets analyzed during the current study are publicly available from the Research Ethics Review Committee Reporting System of the National Institute of Public Health of Japan. The literature data supporting the findings of this study are available from PubMed. Details of data processing, classification procedures, and analytical methods are provided in the supplementary materials.

## Abbreviations

AI: Artificial intelligence
CI: Confidence interval
CRBs: Certified review boards
FDA: Food and Drug Administration
IRB: Institutional review board
MeSH: Medical Subject Headings
MEXT: The Ministry of Education, Culture, Sports, Science and Technology
MHLW: Ministry of Health, Labour and Welfare
PMDA: Pharmaceuticals and Medical Devices Agency
REC: Research ethics committee
RS: Regulatory science
SD: Standard deviation
U.S.: United States
