# Supplementary material for: Structural characteristics of research ethics committees in Japan and their relevance to regulatory science: a nationwide descriptive study
Source: BMC Med Ethics. 2026 Jul 7;27:212. doi: 10.1186/s12910-026-01553-w (PMC13629136; doi:10.1186/s12910-026-01553-w)
Supplement: Supplementary file 1 [file 12910_2026_1553_MOESM1_ESM.zip › BMC_Supplimental_Tables_hara_20260325.docx]

**Supplemental Table 1: Categorization of Individual Professional Fields and Corresponding Keywords**

|  |  |  | Keywords |  |
| --- | --- | --- | --- | --- |
| SCIENCE EXPERT | | | | |
|  | **Experts in Biomedical and Human Research** | | | |
|  |  | Physicians and Dentists | Physicians, dentists, medical specialists, department heads, hospital directors, clinicians, and various clinical departments |  |
|  |  | Nursing and Midwifery | Nursing, midwifery, public health nurses, health and medical care, and patient safety |  |
|  |  | Pharmaceutical Sciences and Industry | Pharmacology, pharmaceutics, drug formulation, pharmacodynamics, drug discovery, pharmacy, vaccines, and related terms |  |
|  |  | Co-Medical and Allied Health Professionals | Rehabilitation, physiotherapy, occupational therapy, sports science, nutrition, chiropractic, acupuncture, preventative health, and related terms |  |
|  |  | Genetics, Genomics, and Molecular Biology | Genetics, genomics, cellular biology, microbiology, virology, biotechnology, RNA research, biochemistry, and molecular biology |  |
|  |  | Non-Genomic Human Biology and Related Fields | Foundational biological sciences and related areas |  |
|  |  | Toxicology and Veterinary Sciences | Toxicology, veterinary science, and experimental animals |  |
|  |  | Reproductive Medicine and Biology | Regenerative medicine, reproduction, stem cell research, embryology, and related fields |  |
|  |  | Diagnostics (In Vivo and In Vitro) | Clinical testing, radiology, pathology, imaging, diagnostic techniques, and related terms |  |
|  |  | Medical Devices and Engineering | Biomedical engineering, clinical engineering, prosthetics, radiological devices, and related technologies |  |
|  |  | Social Medicine and Public Health | Social medicine and related public health policies |  |
|  |  | Epidemiology and Statistics | Epidemiology, biostatistics, public health data, and bioinformatics |  |
|  |  | Clinical Trials and Research | Clinical trials, clinical research, and associated fields |  |
|  | **Experts in Non-Human Science** | | |  |
| humanities and social researcher | | | | |
|  | **Humanities and Social Research (Legal and Psychology)** | | | |
|  |  | Legal and Judicial Affairs | Legal professionals, judiciary, attorneys, court, legal affairs, law, personal data protection, and human rights |  |
|  |  | Psychology, religion, and ethics | Psychology, ethics, religion, spirituality, mental health, philosophy, and related terms |  |
|  | **Humanities and Social Research (Other)** | | |  |
| General public | | | | |

**Supplemental Table 2: Composition of Occupation Categories among Institution categories.**

| Factor | | Hospi. | Univ. | Com. | Clinic | NPO | RI | Medi. Co. | AS | Gov. | NC |
| --- | --- | --- | --- | --- | --- | --- | --- | --- | --- | --- | --- |
| N | | 12826 | 5747 | 2087 | 1765 | 1653 | 886 | 796 | 536 | 326 | 164 |
| Experts In Biomedical and Human Research | | 8339 (65.0) | 3374 (58.7) | 810  (38.8) | 1000  (56.7) | 1032  (62.4) | 457  (51.6) | 471  (59.2) | 383  (71.5) | 146  (44.8) | 108  (65.9) |
|  | Physicians and dentists | 4324  (33.7) | 971  (16.9) | 221  (10.6) | 491  (27.8) | 378  (22.9) | 123  (13.9) | 208  (26.1) | 216  (40.3) | 42  (12.9) | 39  (23.8) |
|  | Nursing and midwifery | 1636  (12.8) | 721  (12.5) | 43  (2.1) | 161  (9.1) | 95  (5.7) | 48  (5.4) | 98  (12.3) | 21  (3.9) | 26  (8.0) | 10  (6.1) |
|  | Pharmaceutical sciences and industry | 1082  (8.4) | 315  (5.5) | 271  (13.0) | 103  (5.8) | 299  (18.1) | 46  (5.2) | 38  (4.8) | 16  (3.0) | 15  (4.6) | 9  (5.5) |
|  | Diagnostics in vivo and in vitro | 439  (3.4) | 175  (3.0) | 37  (1.8) | 48  (2.7) | 24  (1.5) | 15  (1.7) | 34  (4.3) | 3  (0.6) | 6  (1.8) | 6  (3.7) |
|  | Co-medical and allied health professionals | 368  (2.9) | 499  (8.7) | 58  (2.8) | 63  (3.6) | 89  (5.4) | 23  (2.6) | 56  (7.0) | 31  (5.8) | 15  (4.6) | 0  (0.0) |
|  | Non-genomic human biology and related fields | 290  (2.3) | 314  (5.5) | 66  (3.2) | 50  (2.8) | 32  (1.9) | 67  (7.6) | 27  (3.4) | 18  (3.4) | 21  (6.4) | 20  (12.2) |
|  | Social medicine and public health | 222  (1.7) | 96  (1.7) | 23  (1.1) | 28  (1.6) | 56  (3.4) | 27  (3.0) | 14  (1.8) | 67  (12.5) | 11  (3.4) | 2  (1.2) |
|  | Medical devices and engineering | 132  (1.0) | 98  (1.7) | 15  (0.7) | 27  (1.5) | 23  (1.4) | 10  (1.1) | 12  (1.5) | 15  (2.8) | 2  (0.6) | 6  (3.7) |
|  | Genetics genomics and molecular biology | 66  (0.5) | 319  (5.6) | 69  (3.3) | 31  (1.8) | 27  (1.6) | 67  (7.6) | 6  (0.8) | 2  (0.4) | 4  (1.2) | 22  (13.4) |
|  | Clinical trials and research | 47  (0.4) | 5  (0.1) | 4  (0.2) | 7  (0.4) | 4  (0.2) | 8  (0.9) | 1  (0.1) | 0  (0.0) | 0  (0.0) | 4  (2.4) |
|  | Epidemiology and statistics | 33  (0.3) | 148  (2.6) | 9  (0.4) | 8  (0.5) | 34  (2.1) | 45  (5.1) | 1  (0.1) | 8  (1.5) | 17  (5.2) | 7  (4.3) |
|  | Toxicology and veterinary sciences | 5  (0.0) | 11  (0.2) | 14  (0.7) | 0  (0.0) | 1  (0.1) | 8  (0.9) | 1  (0.1) | 0  (0.0) | 0  (0.0) | 0  (0.0) |
|  | Reproductive medicine and biology | 4  (0.0) | 19  (0.3) | 5  (0.2) | 22  (1.2) | 9  (0.5) | 2  (0.2) | 8  (1.0) | 0  (0.0) | 0  (0.0) | 0  (0.0) |
| Experts in non-human science | | 3  (1.8) | 27  (1.6) | 35  (2.0) | 12  (1.5) | 7  (1.3) | 197  (9.4) | 69  (7.8) | 32  (9.8) | 287  (5.0) | 152  (1.2) |
| Humanities (legal and psychology) | | 1167 (9.1) | 959 (16.7) | 344 (16.5) | 262 (14.8) | 238 (14.4) | 129 (14.6) | 118 (14.8) | 72 (13.4) | 54 (16.6) | 29 (17.7) |
|  | Legal and judicial affairs | 829  (6.5) | 418  (7.3) | 303  (14.5) | 199  (11.3) | 184  (11.1) | 89  (10.0) | 89  (11.2) | 51  (9.5) | 38  (11.7) | 10  (6.1) |
|  | Psychology religion and ethics | 361  (2.8) | 558  (9.7) | 50  (2.4) | 75  (4.2) | 65  (3.9) | 45  (5.1) | 31  (3.9) | 24  (4.5) | 17  (5.2) | 19  (11.6) |
| Humanities and social research (other) | | 29  (17.7) | 278  (16.8) | 302  (17.1) | 129  (16.2) | 95  (17.7) | 396  (19.0) | 169  (19.1) | 64  (19.6) | 1434  (25.0) | 1403  (10.9) |
| General public | | 3074  (24.0) | 824  (14.3) | 703  (33.7) | 457  (25.9) | 342  (20.7) | 206  (23.3) | 200  (25.1) | 58  (10.8) | 93  (28.5) | 27  (16.5) |

The abbreviations used are Hospi. for Hospital, Univ. for University, Com. for Company, Clinic for Clinic, NPO for Non-profit Organization, RI for Research Institute, Medi. Co. for Medical Corporation, AS for Academic Society, Gov. for Government, and NC for National Center.

**Supplemental Table 3: Distribution of MeSH Categories**

| MeSH terms | N |
| --- | --- |
| Drug Approval | 52 |
| Clinical Trials as Topic | 23 |
| Antineoplastic Agents | 22 |
| Neoplasms | 21 |
| Research Design | 20 |
| Drug Development | 19 |
| Pharmaceutical Preparations | 19 |
| Drugs, Generic | 18 |
| Drug-Related Side Effects and Adverse Reactions | 16 |
| Biosimilar Pharmaceuticals | 13 |
| Adverse Drug Reaction Reporting Systems | 11 |
| Drug Industry | 11 |
| Product Surveillance, Postmarketing | 11 |
| Device Approval | 10 |
| Government Agencies | 10 |
| Drug-Eluting Stents | 9 |
| Percutaneous Coronary Intervention | 9 |
| Randomized Controlled Trials as Topic | 9 |
| Cell- and Tissue-Based Therapy | 8 |
| Drug and Narcotic Control | 8 |
| Drug Discovery | 8 |
| Clinical Trials, Phase I as Topic | 7 |
| COVID-19 | 7 |
| Drug Labeling | 7 |
| Equipment and Supplies | 7 |
| Regenerative Medicine | 7 |
| Biological Products | 6 |
| Genetic Therapy | 6 |
| Guidelines as Topic | 6 |
| Multicenter Studies as Topic | 6 |
| Antineoplastic Combined Chemotherapy Protocols | 5 |
| Biomarkers | 5 |
| Biopharmaceutics | 5 |
| Breast Neoplasms | 5 |
| Coronary Artery Disease | 5 |
| International Cooperation | 5 |
| Legislation, Drug | 5 |
| Outcome Assessment, Health Care | 5 |
| Patient Selection | 5 |
| Pharmacovigilance | 5 |
| Therapeutic Equivalency | 5 |
